# Transpiration responds linearly to Penman-Monteith reference evapotranspiration and varies genetically, both in individual plants and canopies, in large sorghum and pearl millet panels

**DOI:** 10.1016/j.plaphe.2026.100231

**Published:** 2026-06-03

**Authors:** Laura Grégoire, Jana Kholova, Sunita Choudhary, Rekha Badham, Clémentine Denis, Jeannot Ndione, Oumar Sall, Cheikh Soce, Romiel Badji, Yves Vigouroux, Vincent Vadez

**Affiliations:** a Institut de Recherche pour le Développement (IRD), University of Montpellier, DIADE Research Unit, Montpellier, 34394, France; b International Crops Research Institute in Semi-Arid Tropics (ICRISAT), Patancheru, 502324, India; c Department of Information Technologies, Faculty of Economics and Management, Czech University of Life Sciences Prague, Kamýcká 129, Prague, 16500, Czech Republic; d Centre d’étude Régional pour l'amélioration de l'adaptation à la sécheresse (CERAAS), Thiès, Senegal; e Institut Sénégalais de Recherches Agricoles (ISRA), Bambey, Senegal

**Keywords:** Vapor pressure deficit, Transpiration restriction, Drought, Reference evapotranspiration, Evaporative demand, Climate change, Crops

## Abstract

Genetic variation in the transpiration response to evaporative demand has been mostly studied in controlled environments, individual plants, and small genetic panels. Moving outdoors in field-like situations raises questions about what timeframe to measure transpiration, which metric to represent evaporative demand (vapor pressure deficit, VPD or reference evapotranspiration, ET_ref_), which model to describe transpiration responses (linear slopes or segmented breakpoints), which plant spacing (individual plants, canopies), and which experimental setup for heritable genetic variation.

Transpiration responses to evaporative demand were phenotyped in 467 sorghum and pearl millet inbred lines in three contrasting platforms (high-tech, low-tech, and lysimetric systems) outdoors. Across all experiments, ET_ref_ consistently explained more variation in transpiration response than VPD. In both species, transpiration responses to increasing ET_ref_ were best described by linear relationships. Genotype-specific regression slopes were retained as trait estimates.

In pearl millet, transpiration response slopes and transpiration efficiency exhibited significant genetic variation with high heritability (0.61-0.73) across platforms. In sorghum, trait heritability depended on the platform, with higher heritability in the low-tech. Transpiration efficiency was positively associated with transpiration response slopes in both species, while relationships with root traits revealed species-specific patterns of water use and uptake.

Overall, ET_ref_ was a reliable descriptor of evaporative demand, and linear modelling captured transpiration responses to ET_ref_ well outdoors. The low-tech platform enabled reliable phenotyping of transpiration response to ET_ref_ of individual plants in large germplasm panels while both the high-tech and lysimeter platforms were reliable to measure transpiration response to ET_ref_ in canopies.

## 1 Introduction

Sorghum (*Sorghum bicolor* (L.) Moench) and pearl millet (*Cenchrus americanus* (L.) Morrone) are key staple crops in semi-arid tropical regions, valued for their adaptation to high temperatures and water-limited conditions. However, despite these inherent capacities, their yield stability remains strongly affected by moderate drought (reductions of 20–70% are common) [1]. Among the plant physiological processes underlying drought adaptation, the limitation of transpiration under high evaporative demand has been identified as a key trait to save water [2]. Its potential benefits were first highlighted in a crop simulation study [2], where water saved by limiting transpiration under high evaporative demand was available during the critical grain filling stage and led to yield increases under terminal drought. In addition, simulations predicted this mechanism would increase transpiration efficiency. These hypotheses have motivated extensive experimental research to identify genetic variation for this trait in multiple crops [3], including soybean [4,5], maize [6,7], peanut [8], sorghum [9,10], wheat [11], chickpea [12], cowpea [13], lentil [14], faba bean [15], pearl millet [16], sweet corn [17].

All studies to date consistently report genetic variation in transpiration responses to evaporative demand within evaluated germplasm. However, because they have been mostly conducted indoors in glasshouse or growth chambers, they have used very small germplasm panels, except few studies [18,19]. Hence, they remain insufficient to capture the full extent of diversity within many species to support robust downstream genetic analyses. For instance, previous studies in sorghum have been conducted with panels that did not exceed 30 lines [9,10]. Expanding characterization of plant transpiration response to evaporative demand across large and diverse germplasm panels was therefore a critical step. However, a major bottleneck to such scaling lies in the phenotyping capacity. This was the first objective of this study in which we tested and compared different ways of measuring transpiration responses, with a focus on identifying the most appropriate methods to assess: (i) plant transpiration; (ii) leaf area; (iii) evaporative demand.

Working on this trait in controlled or growth chamber conditions with constant light, the evaporative demand was typically described by the vapor pressure deficit (VPD), which integrates temperature and relative humidity. Although controlled-environment experiments provide precise measurements, they fail to capture field-relevant dynamics, where additional factors of the evaporative demand such as light and wind fluctuate throughout the day and influence plant transpiration [10,20]. Light intensity outdoors is also much higher than in growth chambers. Two studies on transpiration responses used the Penman-Monteith reference evapotranspiration (ET_ref_) as a proxy of the evaporative demand [10,18]. However, in both cases they used relatively long timeframes (>1-7 days) to evaluate transpiration and the evaporative demand, rather than the hour-timeframes of earlier studies that used VPD. This raises the question whether these two studies could have missed any transpiration variation caused by hourly variations in ET_ref_. Here, we hypothesized that shorter timeframes to capture fluctuations in transpiration would provide more heritable genotypic responses.

Most previous studies compared the genotypic differences in the transpiration limitation from the presence or absence of a VPD breakpoint beyond which transpiration would either decrease or remain constant [[4], [5], [6], [7], [8], [9],[11], [12], [13], [14], [15], [16], [17]]. Plant responses have then been quantified using segmented linear models testing for presence/absence of a VPD breakpoint where transpiration response slope significantly changes. This representation was challenged in studies showing that the transpiration limitation could also come in the form of differences in the slopes of the transpiration response to VPD [20,21]. A study with a large maize panel showed that the linear model was predominant and that breakpoints were rarely found, and only found at very high ET_ref_ [18]. Another study showed that all genotypes’ transpiration response could be fitted to a linear model [10]. This discrepancy is important to clarify because a linear or a segmented response of transpiration to the evaporative demand do not merely represent two outcomes of a statistical analysis: they highlight two opposite views on the regulation of stomata. A segmented response would imply that there is a transient partial closure of stomata when the evaporative demand increases. By contrast, a linear response would imply inherent stomatal conductance differences among genotypes. Therefore, one of our objectives here was to test which transpiration response model (linear or segmented) best fitted the data and we did that with large germplasm panels in two crop species to ensure robust conclusions, going beyond the small sorghum panel tested earlier [10].

Lastly, most previous studies on the transpiration limitation trait used plants that were well separated from one another, so that all leaves were exposed to VPD of the above-lying air. However, plants in a field rapidly form a crop canopy and because of boundary and leaf layer resistances, canopy and air VPD can decouple, so that not all leaves are exposed to the VPD of the above-lying air. Only one study measured the transpiration response to the evaporative demand (proxied by ET_ref_) in plants forming a canopy and found genotypic variation [10]. However, this was done with a small germplasm panel of sorghum. In addition, this lysimeter-based protocol used long timeframes (>7days) to measure transpiration and ET_ref_. This may have flattened the day-to-day or hour-to-hour variation in ET_ref_ and could have altered the precision in the assessment of the transpiration responses and our capacity to detect possible rapid changes. These long timeframes could also have been the reason why this study did not find any breakpoint in the transpiration responses.

Therefore, this study raises a number of interrelated questions related to the transpiration response to the evaporative demand, questions that arise from earlier studies which tested these transpiration responses with small germplasm panels indoors, used VPD as the proxy for the evaporative demand, used a linear segmented model, and used individual plants. Here we questioned: (i) which timeframe measures the transpiration responses the most accurately (ii) which proxy is best to represent the evaporative demand, VPD or ET_ref_, (iii) whether genetic variations in the transpiration limitation are best represented by slope differences of linear regressions or by segmented models with a breakpoint,(iv) whether genotypic variation in the transpiration response to the evaporative demand can be assessed in large genotype panels in plants forming a canopy, and finally conclude (v) which protocols are best suited to study these responses in large germplasm panels.

For that purpose, we built up on three earlier studies to address these questions and adapted the protocols therein to address the final question (v). A low-cost pot-based study [22] showed the potential of using short term changes in VPD to elicit transpiration changes of individual plants. This was used to design an outdoor platform and protocol using short timescales for the assessment of the transpiration response to evaporative demand for individual plants and address questions (ii) and (iii) above. An outdoor study [23] demonstrated the potential of a high-throughput platform to measure plant transpiration and leaf area. Here we developed a protocol based on this high-throughput platform to measure the transpiration response to VPD and ET_ref_, at timescales of few hours, allowing to address questions (ii) (iii) and (iv) above for plants forming a canopy. This protocol on high-throughput platform was compared to the lysimeter-based protocol of another study that used larger timescales to measured transpiration and ET_ref_ [10], allowing to test question (i) on the adapted timescale for plants forming a canopy (iv). Finally, additional traits were also measured (roots, transpiration efficiency) to test their putative relationship with the transpiration response trait.

## 2 Materials and methods

### 2.1 Phenotyping platform descriptions

**Fig. 1:**
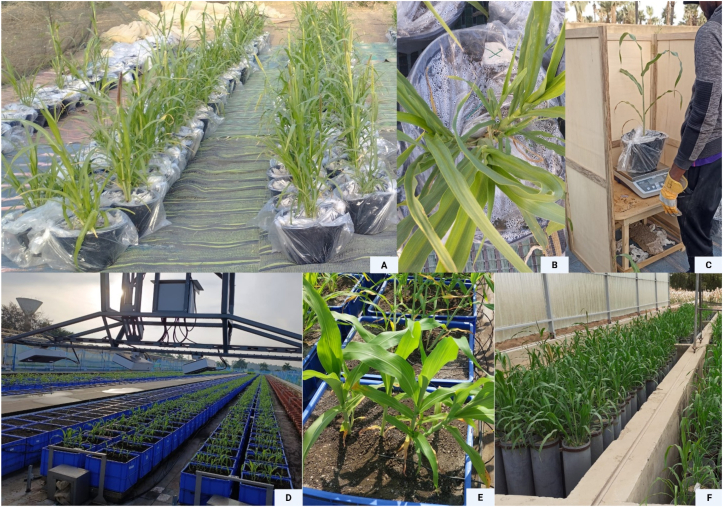
Overview of the three experimental systems used in the study: Low-tech System (A-C), High-tech System (D-E), and Lysimetric System (F). Individual plants are cultivated in buckets arranged in rows (A), wrapped in plastic bags to reduce soil evaporation (B). Buckets are manually weighed four times a day during four days around 50 DAS, with a scale protected from wind and sunlight (C). A PlantEye 3D scanner in the high-tech system captures 3D leaf area twice daily (D). Blue trays with 4 plants per tray lay (forming a crop canopy) on load cells that log tray weight every 15 min (E). Plants in the lysimeter system are cultivated individually in each tube, although they rapidly form a crop canopy at a field-representative density with the neighbouring plants (F).

Three distinct platforms were employed to evaluate this transpiration response trait across five experiments using sorghum and pearl millet panels, each with specific experimental setups regarding the timeframe to measure transpiration (hours vs days), ways to measure leaf area (manual vs automatic) and plant spacing (individual vs canopy). An overview of these systems is presented in Fig. 1. The first was a low-tech phenotyping platform trial utilizing pot weighing to characterize the transpiration response in individual plants, measured over four-time intervals per day, each of a few hours (Fig. 1A–C). Additionally, root traits, including the root-to-shoot ratio and root area, were measured at harvest. Experiments were carried out at the Centre d’Etude Régional pour l’Amélioration de l’Adaptation à la Sécheresse (CERAAS) in Thiès, Senegal (14° 47′N; 16° 56′W). The second (Fig. 1 D, E) was the high-tech LeasyScan platform [23], at the International Crops Research Institute for the Semi-Arid Tropics (ICRISAT) in Hyderabad, India (17°30′N; 78°16′E). This platform used large trays (60x40 × 30 cm, LxWxH) of 0.25 m^2^ filled with local vertisol (containing at least 40% clay) in which 4 plants were grown and formed canopy at typical field densities. Transpiration was inferred from load cell measurements of tray weight, using the same time windows as in the low-tech experiment, and leaf area assessed twice a day with a 3D laser scanner (Phenospex Ltd, Herleen, The Netherlands). The third platform (Fig. 1F) used a lysimeter setup [10] at the Centre National de Recherche Agronomique (CNRA) in Bambey, Senegal (14° 41′N; 16° 27′W). Plants were grown in large lysimeters (150 cm deep, 25 cm diameter). The cylinders were placed side by side, so that the shoots of individual plants quickly formed a continuous canopy, at a plant population of approximately 11 plants m^−2^. Transpiration was inferred from lysimeter weighing every 4 to 7 days. Transpiration efficiency at plant level (TE_plant_) was also measured at the end of the experiment, as the ratio of the aboveground biomass at harvest to the cumulated transpiration.

### 2.2 Plant material

Sorghum material consisted of 240 inbred genotypes from the Generation Challenge Program reference set [24] and were common to the three trials. Given the larger capacity of the LeasyScan platform, the number of accessions was expanded to 320. For the low-tech experiment in pearl millet, the plant material comprised 227 inbred genotypes from the PMiGAP collection, i.e. the Pearl Millet Inbred Germplasm Association Panel 25 , 220 of them were also included in the pearl millet lysimeter study.

### 2.3 Developing a low-tech protocol with sorghum and pearl millet plants

For the low-tech experiment conducted in Thiès, Senegal, plants were grown individually in 10L buckets filled with a typical Sahelian sandy soil, composed of over 90% sand, complemented with dry cow manure, sieved prior to sowing and incorporated at a rate of 400 g per bucket. In 2024, the panel of 240 sorghum genotypes was divided into two batches. The first batch was sown on 4 December 2023, transpiration measured on 29-30-31 January and 01 February, and plants harvested on 2 February 2024. The second batch was sown on 18 December 2023, transpiration measured on 12-15 February, and plants harvested on 16 February 2024. For pearl millet, the panel of 227 genotypes was similarly divided into two batches. The first batch was sown on 16 December 2024, transpiration measured 3-6 February, and plants harvested on 7 February 2025, whereas the second batch was sown on 30 December 2024, transpiration measured 17-20 February, and plants harvested on 21 February 2025. No genotypes were duplicated between the two batches in the sorghum trial, with 120 genotypes per batch and four replicates per genotype. In contrast, for the pearl millet trial, 13 pearl millet genotypes out of the total of 227 were included in both batches (120 genotypes in the first batch and then 107 + 13 genotypes in the second batch), with four replicates per genotype in each batch (these genotypes with a total of eight replicates were excluded of the following analysis to avoid unbalanced dataset). The two planting dates were considered as batches of a single low-tech experiment rather than independent experiments, as they were conducted under similar experimental conditions (location, soil, management practices, measurement protocol at same development stage, and comparable environmental conditions between batches, Figs. S1–S3). To account for potential batch effects, the factor “batch” was included in the analysis and tested as a random effect (see Statistical Analysis section).

The experimental setup in each batch consisted of 480 buckets, divided in eight columns of 60 buckets in both trials (Fig. 1A). A replicated alpha-lattice design was implemented, with 30 genotypes (two columns of 15 buckets) per block for sorghum. The pearl millet trial used the same experimental design. All experimental designs described in this study were implemented using the R package FieldHub [26]. Sowing was conducted at a rate of three hills per bucket, with five seeds per hill. Two thinning events were carried out: for sorghum, at 10 and 21 days after sowing (DAS), and for pearl millet, at 15 and 25 DAS. The first thinning left one plant per hill and after the second thinning one plant per bucket was retained. Irrigation was initially provided at a rate of 0.5 L every 2–3 days, increasing to 1 L as the plants grew larger.

On the 54th and 47th DAS for the sorghum and pearl millet experiments, respectively, buckets were brought to field capacity by abundant watering and overnight drainage. The following morning, buckets were enclosed in plastic bags that were wrapped around the plant stem to minimize soil evaporation (Fig. 1B). Measurements were performed for four consecutive days from 56 to 59 DAS for sorghum and from 49 to 52 DAS for pearl millet. During this period, plants were weighed manually four times daily at fixed intervals: 8:00, 12:00, 15:00, and 17:30 to capture a broad range of ET_ref_ values throughout the day. This gave 15 transpiration data points per replicate plant in both sorghum and pearl millet. The scales were shielded from wind and sunlight using a folding screen (Fig. 1C). Transpiration (g.h^−1^) was calculated from the weight difference between consecutive measurements divided by the time interval. Measurement dates were chosen to ensure plants had reached a sufficiently large size in vegetative stage, thereby ensuring large transpiration rate values and thus improving the intensity of the transpiration signal relative to experimental error. During the measurement period, plants received irrigation at the end of the second day (end of 57 and 50 DAS for sorghum and pearl millet). Quantities applied (0, 250, 500, or 750 mL) were calculated on the basis of their transpiration measured during the two days, so that they were re-watered to approximately 90% field capacity and would not experience water stress during the remaining two days of measurement.

Harvest was performed at 60 DAS for sorghum and 53 DAS for pearl millet. Leaf area (cm^2^) was evaluated destructively with a leaf area meter (Li3000, LICOR, Lincoln, Nebraska, USA). Transpiration values measured were all normalized by this value of leaf area at harvest (giving a transpiration rate, TR, in g.h^−1^.cm^−2^). Harvesting also included root system measurements. For sorghum in 2024, the procedure was performed for only two replicates, while for pearl millet in 2025, all replicates were measured. After washing, a subsample of the cleaned root system was collected, by sampling all the roots from two crown roots to ensure the sample was representative of the root system. The root surface area was measured with WinRhizo Root Analysis Software (Regent Instruments Inc., Canada). This subsample was then dried after scanning, as well as the rest of the root system, to determine the root surface area to root dry weight relationship, which was subsequently applied to estimate the total root surface area of the plant (in mm^2^). Shoot samples were also dried for 3 days at 50 °C in a greenhouse, followed by 1 day at 60 °C in an oven, and then weighed. The root-to-shoot biomass and the root/shoot area ratio were then computed.

### 2.4 Developing a high-tech protocol on a large sorghum panel

The high-tech study was conducted at the LeasyScan platform in Hyderabad, India [27], and was only carried out with the sorghum panel of 320 genotypes. Large trays placed on automatic scales were distributed in eight columns ∗ 160 rows. The experiment was designed as an alpha lattice in 8 blocks perpendicular to the column, with 40 trays per block and 4 replicates. Plants were sown in each tray on 14 September 2023. 132 plants were transplanted at 5 DAS and 44 were reseeded at 12 DAS due to germination problems. The final thinning was carried out at 13 DAS, leaving four plants laid in a single row in each tray, giving a plant population of about 16 plants m^−2^, so that the above-ground part of the plants in this experiment quickly formed a crop canopy and remained at the vegetative stage for the duration of the experiment (Fig. 1E). The soil was fertilized with diammonium phosphate at the time of sowing at a rate of 6 g per tray. Each tray was fully irrigated throughout the experiment using a drip irrigation system, every 3-4 days at around 15:00, until field capacity was reached.

The measurement period studied here ran from the day after the last thinning to the day before harvesting, from 14 to 33 DAS, with automated weight measurements taken every 15 min. During the measurement period, the crop received six irrigations. As irrigation disrupted the transpiration profiles on those days, irrigation days were excluded from the analysis. Consequently, the analysis was conducted over a span of 14 full days, between 14 and 33 DAS, in which no irrigation was carried out, while ensuring that plants did not experience water stress. Harvest took place between 34 and 37 DAS. Two plants out of 4 for each tray were harvested at 34 DAS (shoot part only). Leaf area was measured on one of the two plants, and stem and leaves weight of that plant were determined after drying in a forced-air oven at 60 °C for 4 days. The remaining two plants were harvested at 37 DAS, and were treated the same way. The purpose of these two samplings was to obtain an estimate of the observed leaf area at 34 and 37 DAS, assuming the leaf area of the harvested plants was similar to that of remaining plant.

As an initial step in data analysis, Kar et al. [28] developed a pipeline for the LeasyScan platform to generate evapotranspiration profiles using weight difference and calculate VPD and ET_ref_ using meteorological data over time. This pipeline was further refined and all modifications and proposed enhancements are available in a public GitHub repository (link in Supplementary Materials). An important step in that pipeline was the estimation of plant transpiration (Tr, mm.15min^−1^) from the evapotranspiration (ET_r_) data that were measured in the platform. This was done via the Novák equation [29]:

$$Tr=\left(1-e^{-\beta \times LAI}\right)\times ET_{r}$$

where β = 0.463 represents a light extinction coefficient for most agricultural canopies [30]. For this, the leaf area index (LAI) was needed. Digital 3D leaf area (mm^2^) was measured by the PlantEye scans conducted twice daily throughout the measurement period; the daily median was used to obtain estimates for each tray from 14 to 25 DAS. Values up to 25 DAS were converted to observed leaf area using the empirical 3D leaf area vs observed leaf area relationship for pearl millet described by Vadez et al. [23], as the corresponding relationship for sorghum remains unpublished. Beyond 25 DAS, 3D leaf area measurements became unreliable due to leaf overlap (leading to underestimation) and barcode recognition errors (resulting in missing data). To address this, the leaf area values up to 25 DAS and the leaf area sampling at 34 and 37 DAS were plotted over time to interpolate missing or noisy values between 27 DAS and the harvest, assuming a linear daily leaf area increase during the vegetative stage. From this, a leaf area index (LAI) was then computed for each day from 14 to 33 DAS and used to estimate plant transpiration (Tr, mm.15min^−1^) from evapotranspiration (ET_r_) data with the Novák equation [29]. With these Tr data collected at 15 minute intervals, transpiration (in mm.h^−1^) values were aggregated into the same daily periods as those used in the low-tech trial (08:00–12:00, 12:00–15:00, 15:00–17:30, and 17:30–08:00) and divided by the time interval. Over the 14-day measurement period, this resulted in four time-intervals per day, except on the final day with three intervals, yielding a total of 55 transpiration intervals.

### 2.5 Adapting a lysimeter protocol on large sorghum and pearl millet panels

The third experimental platform involved a lysimeter setup established in Bambey, Senegal. Field trials were conducted outdoors for both sorghum (2024) and pearl millet (2025).

The experimental units were arranged within eight trenches, each containing 20 rows with six tubes per row, 120 tubes per trench, totalling 960 tubes. Both experiments followed an alpha-lattice design. In the sorghum trial, the design comprised 32 incomplete blocks oriented perpendicular to the trench length, each block containing 30 genotypes. Most genotypes were replicated four times, while 24 genotypes had only three replicates due to seed constraints; the missing fourth replicates were treated as missing data during analysis. In total, 936 PVC tubes were used in the sorghum data analysis. The pearl millet trial included 228 genotypes, of which 36 were replicated four times and the remaining 192 were replicated three times, resulting in 720 experimental units. The alpha-lattice design then consisted of 24 incomplete blocks distributed across four replications, with each block comprising 38 units. Data from this design, were analysed using the FieldHub package in R. A mixed linear model was fitted using the restricted maximum likelihood method, with genotype treated as a fixed effect and replication and incomplete block as random effects. Missing data were directly accounted for within the REML framework, which estimates parameters using all available information without requiring data imputation [26].

Sowing of the sorghum panel took place over two days (22–23 January 2024), with a seeding rate of two hills per tube and five seeds per hill. Pearl millet was sown in a single day on 17 March 2025 using the same method. Thinning was performed for sorghum at 10 and 14 days for both crops, leaving one healthy plant per tube after the last thinning. Fertilizer (NPK 15-15-15) was applied following each thinning event at a rate of 2 g per tube. An insecticide treatment (Bomec) was applied at a concentration of 2 mL per 10 L of water for the sorghum trial. Prior to sowing and before initiating transpiration measurements, the tubes were watered up to field capacity after thorough irrigation and drainage. Weighings started at 23DAS for both crops. A pulley system connected to an S-type load cell (Mettler-Toledo, Geneva, Switzerland) enabled regular individual weighing of each tube. The initial weight at 23 DAS for both crops was considered to be the field capacity weight, which was used throughout the experiment to re-water the cylinders. Soil evaporation was limited by the addition of a 5 cm layer of gravel on top of the tubes. Transpiration was then estimated from the consecutive weighing of cylinders and from the water addition after each weighing. After each weighing, tubes were re-watered to approximately 90% of their field capacity.

The sorghum and pearl millet trials lasted 13 and 9 weeks, respectively, with final harvests conducted on 6 April 2024 and 23 May 2025. During these periods, 18 weighings were conducted for the sorghum trial and 14 for the pearl millet trial, corresponding to 17 and 13 transpiration time intervals. At harvest, aboveground biomass was collected and separated into stem, leaf and panicles. Total biomass was recorded after oven drying at 60 °C for three days. Plant-level transpiration efficiency TE_plant_ (g·kg^−1^) was calculated as the ratio of total aboveground biomass (g) to cumulative transpiration (kg) during the weighing period. There, it was assumed that the initial biomass when weighing started was negligible compared to the final biomass.

### 2.6 Calculation of VPD and reference evapotranspiration ET_ref_

In each trial, transpiration for each time interval was paired with the corresponding ET_ref_ and mean VPD. Weather data were recorded at 15-min intervals for the high-tech experiment and at 30-min intervals for the low-tech and lysimeter trials. The mean vapor pressure deficit (VPD, kPa) was calculated with the air temperature (T, °C) and relative humidity (RH, %) as following

$$VPD=0.6108\times \exp\left(\frac{17.27\times T}{T+237.3}\right)\times \left(1-\frac{RH}{100}\right)$$

over defined time windows (e.g., 08:00–12:00, 12:00–15:00, …) for the high-tech and low-tech experiments, or between consecutive weighing events for the lysimeter setup. Reference evapotranspiration (ET_ref_) was estimated using the Penman–Monteith equation, at the time frame of the weather data collected in each platform (15- or 30-min interval). ET_ref_ values were then aggregated to obtain cumulative ET_ref_ over each corresponding interval. ET_ref_ was expressed in mm·h^−1^ for the high-tech and low-tech experiments and in mm·day^−1^ for the lysimeter measurements.

The Penman–Monteith formulation incorporated net radiation (Rn – G, converted from W/m^2^ to MJ·m^−2^·h^−1^), mean hourly wind speed ($W_{s}$, converted from km·h^−1^ to m·s^−1^), mean air temperature (T, °C), vapor pressure deficit ($e_{s}-e_{a}\text{,}$ kPa), the slope of the saturation vapor pressure curve (Δ, kPa·°C^−1^), and the psychrometric constant (γ = 0.0665 kPa °C^−1^).

$$ET_{\text{ref}}=\frac{0.408\Delta \left(\text{Rn}-G\right)+\gamma \left(\frac{900}{37}\right)\frac{1}{T+273}W_{s}\left(e_{s}-e_{a}\right)}{\Delta +\gamma \left(1+0.34W_{s}\right)}$$

The meteorological data for each trial are presented in the supplementary material (Figs. S1–S3). For the pearl millet lysimeter trial in 2025, the main weather station connected to the lysimeter platform was non-operational during the experiment. Consequently, daily records of temperature, relative humidity, and wind speed were obtained from an auxiliary on-site station. Solar radiation data were sourced from the nearest (about 15 km away) functioning station located in Niakhar (14°28′60″ N, 16°24′0″ W) on a 30 min interval. In the pearl millet trial, a rain shelter had been installed on top of the lysimeters and decreased radiation by about 50%. Hence, a correction coefficient of −50% was applied to solar radiation data in the computation of ET_ref_. This coefficient was derived from manual solar radiation measurements conducted at the trial site under the shelter and outside the shelter.

### 2.7 Selection of transpiration response variables and evaporative demand descriptors

To address question (ii) of the introduction, three descriptors of the evaporative demand explanatory variables (X) of the transpiration response were considered: VPD, ET_ref_, and ET_ref_ adjusted by a crop coefficient K_c_ that reflected the possible changes in the leaf area index over the course of the experiment [31]. In the low-tech context, ET_ref_ × K_c_ was not tested because the four-day measurement period was too brief to consider any significant changes in the LAI that would have needed a correction by a crop coefficient. As for the transpiration responses (dependent variable Y) two metrics were used, depending on plant spacing. For the low-tech measurements, plants were grown individually and were well separated from one another, so that all their leaves were exposed to the evaporative demand of the air, the response variable was transpiration normalized by the leaf area (transpiration rate, TR, in g.h^−1^.cm^−2^). In the lysimeter and high-tech platform, plants formed a canopy and top and bottom leaves were likely exposed to different evaporative demand. Therefore, the response variable was transpiration of the canopy and data were not normalized by the leaf area (Tr, in mm.h^−1^ or mm.d^−1^), as was previously done in a similar lysimeter setup [10].

To address question (iii) of the introduction, both linear and segmented regression models were fitted for each genotype × replication across all combinations of transpiration response and explanatory variables. Model performance was evaluated using the Akaike Information Criterion (AIC) and the root mean square error (RMSE); coefficients of determination (R^2^) were also calculated for descriptive purposes. In addition, regression outputs were visually inspected for each genotype to assess biological plausibility. Once the selected variables and regression were determined for each trial, the parameters of the selected regression (the slope in case of linear regression, the slopes and breakpoint in segmented regression) were retained as trait descriptors for subsequent analyses.

### 2.8 Statistical analysis

For the total of 11 traits measured in the sorghum and pearl millet experiments (summarized in Table 1), outliers were treated using the Tukey boxplot method. For each trait, values falling outside 1.5x interquartile range (Q1 – 1.5 IQR and Q3 +1.5 IQR) were removed and considered as missing values. Histograms were generated for each trait to evaluate distribution patterns, and descriptive statistics were computed. One-way ANOVA was applied to test the effects of the structure and experimental dates on trait variation (block, repetition, row, column, plus batch for low-techs and sorghum lysimeter contexts) with significant factors subsequently incorporated as random effects. Linear mixed models were fitted using the R package lme4 [32] treating genotype as a random effect along with the previously identified random terms. Heritability was calculated using Cullis estimation methods [33] to deal with possible unbalanced datasets following the formula $H^{2}Cullis=1-\frac{\;{\overset{®}{v}\Delta }^{\text{BLUP}\;}}{2\times \sigma_{g}^{2}\;}$ where σ^2^ refers to variance, g to genotype, $\overset{®}{v}$ Δ ^*BLUP*^ to the average standard error of the genotypic Best linear Unbiased Prediction (BLUP). Trait means were calculated per genotype, and Pearson correlation coefficients were computed to evaluate the consistency of transpiration responses to evaporative demand across platforms, as well as their correlations with TE_plant_ and root traits, visualised using the R package corrplot. The BLUPs were calculated for each trait, for subsequent genetic analyses.

**Table 1:** Trait description measured in five trials on three phenotyping platforms. Traits were measured in the low-tech, high tech and lysimeter platforms for both pearl millet and sorghum crops, with a brief summary of the main differences among platforms (soil texture, plant spacing, transpiration measurements and time interval between weighing measurements).

Trial	Species	Soil	Plant spacing	Trait	Time interval	Description
**Low-tech**	**Sorghum and Pearl millet**	**Sandy**	**Individual plant**	Descriptors of linear or segmented regression	Manual weight measurements at 08:00, 12:00, 15:00 and 17:30 during 4 days ( ± 50 DAS)	Relationship between plant transpiration normalized by leaf area (g. h^−1^.cm^−2^), and evaporative demand (‘either VPD, kPa, or ET_ref_, mm.h^−1^)
				Root Area (2 repetitions sorghum, 4 repetitions pearl millet)	-	Root surface measured (Winrhizo) on a subset of 2 roots and estimated on the entire root system based on subset weight/surface ratio.
				Root–Shoot ratio	-	Ratio of root dry biomass and shoot dry biomass
						
**High-tech**	**Sorghum**	**Clay**	**Canopy**	Descriptors of linear or segmented regression	Automated 15-min weight measurements aggregated into the same low-tech intervals over 14 days from 14 to 33 DAS	Relationship between whole-plant transpiration (mm.h^−1^) and evaporative demand (either VPD, kPa, or ET_ref_ or ET_ref_ ×K_c_, mm.h^−1^)
						
**Lysimeter**	**Sorghum and Pearl millet**	**Sandy**	**Canopy**	Descriptors of linear or segmented regression	Measurements several days apart (4 to 7 days) during crop cycle (23–91 DAS)	Relationship between whole-plant transpiration, (mm·day^−1^), and evaporative demand (either VPD, kPa, or ET_ref_ or ET_ref_ ×K_c_, mm.day^−1^)
				TE_plant_	-	Ratio of total shoot biomass at harvest and sum of transpiration during weighing period

## 3 Results

### 3.1 Shorter timeframes capture larger evaporative demand variation

**Fig. 2:**
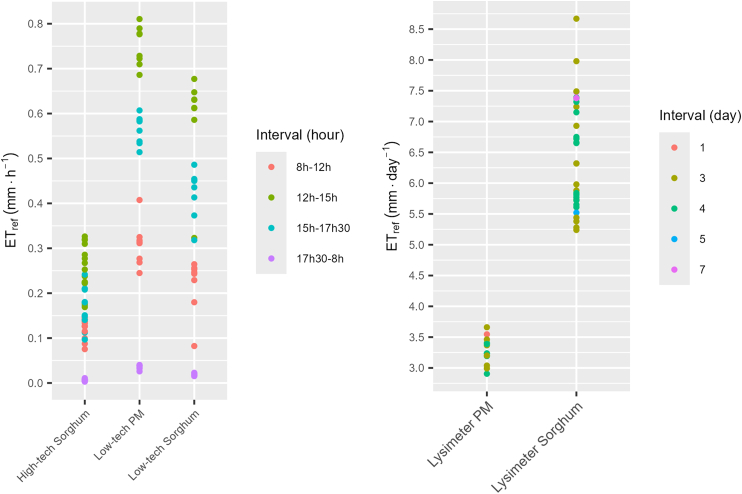
Ranges of reference evapotranspiration (ET_ref_) across five trials on three phenotyping platforms (low-tech, high-tech, and lysimeter system). ET_ref_ is shown for high-tech and low-tech platforms (measurement intervals 08:00–12:00, 12:00–15:00, 15:00–17:30, 17:30_08:00; mm.h^−1^; left) and for lysimeter trials (intervals 1–7 days; mm.day^−1^; right). Pearl millet (PM) and sorghum were evaluated in low-tech and lysimeter trials, while only sorghum was assessed in high-tech trial. Measurement intervals were selected to capture a broad range of ET_ref_ and diverse transpiration responses.

Among the five experiments, a broad range of evaporative demand conditions was observed, with ET_ref_ values recorded across trials are shown in Fig. 2. In the low-tech platform (and to a lesser extent in the high-tech platform), short measurement intervals enabled the capture of a wide range of ET_ref_ values. Repeated measurements throughout the day allowed the detection of near-zero ET_ref_ during evening-night time transpiration (17:30–08:00; purple points in Fig. 2, left) as well as peak ET_ref_ during midday (12:00–15:00; green points). Although the high-tech platform exhibited a narrower ET_ref_ range (0.003 - 0.33 mm h^−1^) than the low-tech sorghum (0.02–0.68 mm h^−1^) and pearl millet trials (0.03–0.81 mm h^−1^), it followed the same diurnal pattern of variation across measurement intervals. The high-tech dataset comprised 55 measurement intervals compared with 30 intervals of measurement in the low-tech trials. Consequently, despite the narrower ET_ref_ range, the large number of observations in the high-tech trial supported the robustness of the results. In contrast, the lysimeter experiments (Fig. 2, right) relied on less frequent weight measurements, with intervals between consecutive weighing ranging from 4 to 7 days. During the 2024 sorghum trial, ET_ref_ ranged from 5.24 to 9.67 mm·day^−1^. The pearl millet trial showed a substantially narrower ET_ref_ range (2.91–3.66 mm·day^−1^), likely because of the presence of the rainout shelter in fixed position on top of this trial.

Differences in ET_ref_ cited above can be explained by specific weather variable difference year to year. For example, ET_ref_ ranges observed between sorghum and pearl millet lysimeter trials can be attributed (in addition to the rainout shelter) to variation in solar radiation, whereas mean temperature and relative humidity were similar between years (Supplementary Figs. S1–S3). ET_ref_ contrasted between high-tech and low-tech trials, particularly the narrower range in the high-tech trial. The high-tech trial, conducted in India in September-October, was characterized by a higher mean relative humidity and a lower cumulative solar radiation, while mean temperature remained within the same order of magnitude, compared to the low-tech trials conducted in Senegal in February (Supplementary Figs. S1–S3). Overall, the broad range of ET_ref_ values across experiments ensured sufficient variation in evaporative demand to elicit contrasting transpiration responses among genotypes in the panel. Nevertheless, it is worth noting that the measurement intervals (and thus the time between two measurements) have an impact on this range: the shorter duration applied for low-tech (and high-tech) trials allowed measurements to be taken under a wider variety of weather conditions than the longer timeframe used in the lysimeter, and was therefore suitable for capturing the larger range of reference evapotranspiration.

### 3.2 ET_ref_ outperforms VPD as descriptor of the evaporative demand

Concerning the variables to represent the evaporative demand related to question (ii), either VPD, ET_ref_ and ET_ref_ × K_c_, the result was globally similar among species and platforms (Table 2). On the low-tech platform for sorghum, the relationship between normalized transpiration and ET_ref_ produced very high mean R^2^ values (0.96, range 0.66-0.99), accompanied with lower median AIC values (−317 compared to −301) extremely low RMSE (≈1 × 10^−5^). Models based on VPD performed marginally less well (mean R^2^ = 0.92, range 0.61-0.98). A similar pattern was observed for pearl millet in the low-tech experiment, where ET_ref_ again provided a slightly better fit compared with VPD (mean R^2^ = 0.94 vs 0.91) and a lower AIC (−283 vs −275) (seeTable 2 ).

**Table 2:** Descriptive statistics of the linear regression analyses between transpiration and the evaporative demand. Either transpiration (Tr) or transpiration normalized by leaf area (transpiration rate, TR) was used as the dependent variable Y and three proxies of the evaporative demand as explanatory variable X (either ET_ref_, ET_ref_ x Kc, or VPD). The bold and italic row indicates the variable selected for each setup based on superior model performance (higher R^2^ and/or lower AIC and RMSE). While ET_ref_ seems to be slightly more efficient to describe evaporative demand in sorghum lysimeter context, ETref ∗ K_c_ was retained for comparison with pearl millet.

Trait	Y	X	Min R^2^	Max R^2^	Mean R^2^	Median AIC	Median RMSE
**Sorghum**							
**Low-tech**	**TR**	***ET***_***ref***_	***0.66***	***0.99***	***0.96***	***−317***	***0.00001***
		VPD	0.61	0.98	0.92	−301	0.00001
**High-tech**	**TR**	***ET*_*ref*_**	**0.023**	*0.96*	***0.81***	***-151***	***0.04843***
		ET_ref_ x K_c_	0.004	0.95	0.77	-140	0.05441
		VPD	0.005	0.88	0.72	-128	0.06198
**Lysimeter**	**Tr**	***ET*_*ref*_*×K_c_***	***0.03***	***0.97***	***0.70***	***−551***	***0.14100***
		ET_ref_	0.08	0.96	0.72	−590	0.13500
		VPD	0.20	0.92	0.65	−424	0.16000
**Pearl millet**							
**Low-tech**	**TR**	***ET*_*ref*_**	***0.58***	***0.99***	***0.94***	***−283***	***0.00001***
		VPD	0.47	0.98	0.91	−275	0.00001
**Lysimeter**	**Tr**	***ET*_*ref*_*×K_c_***	***0.24***	***0.96***	***0.80***	***−458***	***0.16068***
		ET_ref_	0.23	0.94	0.73	−198	0.20184
		VPD	0.20	0.96	0.68	−73	0.22446

In the sorghum high-tech experiment, canopy transpiration showed a strong relationship with ET_ref_, with the highest mean R^2^ (0.81; range: 0.023–0.96, Table 2) and the lowest median AIC (−151) and RMSE (0.04843) among the three models. VPD-based model did not perform well in high-tech and remained inferior to ET_ref_ -based models, with a lower mean R^2^ (0.72), a higher AIC (−128) and higher RMSE (0.06198).

In lysimeter experiments with the sorghum panel, canopy transpiration was best explained by ET_ref_, which yielded a mean R^2^ of 0.72 (range: 0.08–0.96), along with lower median AIC (−590) and RMSE (0.135) values, hence with slightly higher R^2^ than ET_ref_ × K_c_ (mean R^2^ = 0.70) and VPD (mean R^2^ = 0.65). Although VPD exhibited a higher minimum R^2^ (0.20), its higher AIC (−424) and RMSE (0.160) indicated inferior model performance. With pearl millet under lysimeter conditions, the ET_ref_ × K_c_ model provided the best fit, with a mean R^2^ of 0.80 (range: 0.24–0.96), outperforming ET_ref_ alone (mean R^2^ = 0.73) and VPD (mean R^2^ = 0.68). This model was also associated with lower median AIC (−458) and RMSE (0.16) values than the other tested combinations.

Therefore, across experiments, ET_ref_ and ET_ref_ × K_c_ provided the most robust proxy to represent evaporative demand, whereas VPD generally resulted in lower model performance, particularly in lysimeter experiments. Accordingly, ET_ref_ was selected as the primary descriptor for most trials. Because ET_ref_ × K_c_ yielded superior performance for pearl millet under lysimeter conditions and performed similarly to ET_ref_ for sorghum, ET_ref_ × K_c_ was ultimately retained as the evaporative demand descriptor for the sorghum lysimeter trial to facilitate the comparison between species.

### 3.3 A linear model best represents transpiration responses to ET_ref_ across experimental setups

**Fig. 3:**
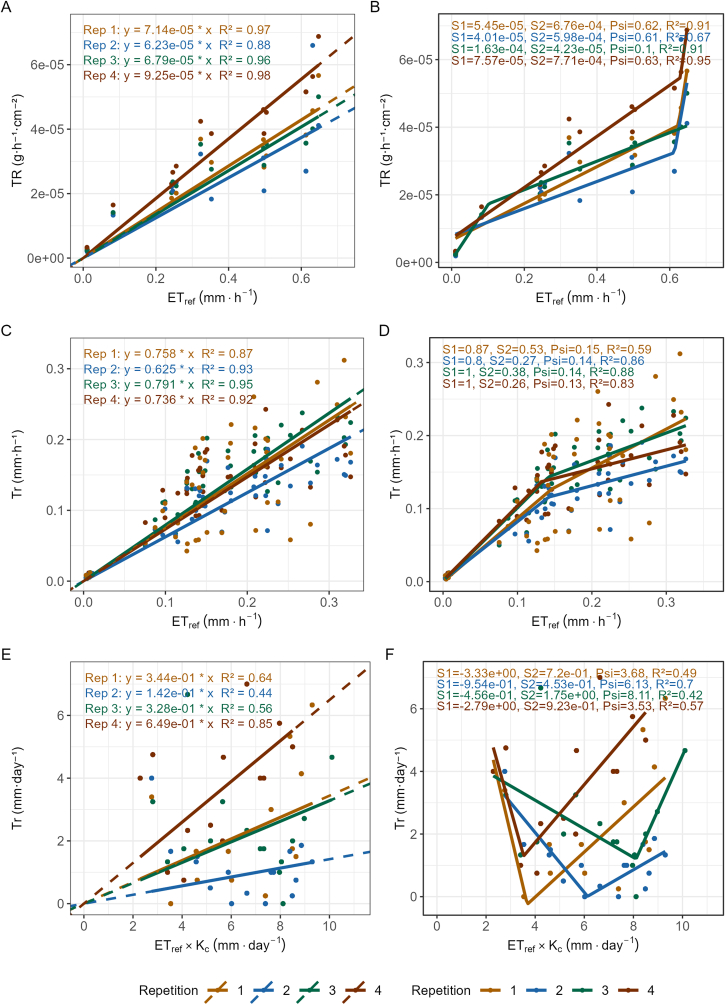
Transpiration responses to evaporative demand for the sorghum genotype IS11119 across three phenotyping platforms: low-tech (A–B), high-tech (C–D), lysimeter system(E–F). Linear (left panels) and segmented (right panels) regressions were fitted separately for each repetition to quantify the relationships between canopy transpiration (mm. h^−1^ in high-tech and mm.day^−1^ in lysimeter) or leaf-area-normalized transpiration rate (TR, g.h^−1^.cm-^2^) against ET_ref_ (mm.h^−1^ in high-tech and low-tech platforms) or ET_ref_ adjust with a crop coefficient K_c_ (mm.day^−1^ in lysimeter).

Fig. 3 illustrates the transpiration response to the evaporative demand for a representative sorghum genotype in the panel, with linear and segmented regressions, showing patterns observed in low-tech (Fig. 3A–B), high-tech ( Fig. 3C–D), and lysimeter trials (Fig. 3E–F) to examine question (iii).

In the low-tech trial, both regressions performed well, although the linear segmented model in Fig. 3B made little biological sense. Also, R^2^ ranging 0.67–0.95 in the segmented regression were slightly lower than in the linear regressions (0.88–0.98), which also had consistently lower RMSE (Table 3). For the high-tech trial, linear regressions consistently produced higher R^2^ values (0.87–0.95) than segmented regressions (0.59–0.88), with comparable RMSE. The AIC tended to be higher with segmented than with linear regressions, although it had higher RMSE (Table 3). The difference between the two regressions was more noticeable in lysimeter trials, where linear regressions consistently achieved higher R^2^ values across repetitions (0.44–0.85) than segmented regressions (0.42–0.70) with comparable RMSE, and which also made little biological sense. Segmented models occasionally yielded lower AIC but frequently produced physiologically implausible transpiration patterns, such as inverted breakpoints (Fig. 3F). Thus, despite occasional improvements in statistical metrics, segmented regression was deemed biologically unreliable.

**Table 3:** Transpiration responses to the evaporative demand for a representative sorghum genotype. Transpiration responses were tested for genotype IS11119 across three phenotyping platforms: low-tech, high-tech, and lysimeter for each repetition in each trial using a linear or a segmented regression. Table summarize model performance: coefficient of determination (R^2^), Akaike Information Criterion (AIC), and root mean square error (RMSE).

Trial	Regression	Repetition	R^2^	AIC	RMSE
**Low-tech**	**Linear**	1	0.97	−335	5.99E-06
		2	0.88	−298	1.02E-05
		3	0.96	−337	5.79E-06
		4	0.98	−336	5.79E-06
	**Segmented**	1	0.91	−341	4.22E-06
		2	0.67	−298	8.50E-06
		3	0.91	−343	3.87E-06
		4	0.95	−342	4.04E-06
**High-tech**	**Linear**	1	0.87	−-144	5.35E-02
		2	0.93	-230	2.88E-02
		3	0.95	-210	3.33E-02
		4	0.92	-181	3.81E-02
	**Segmented**	1	0.59	-140	5.22E-02
		2	0.86	-254	2.20E-02
		3	0.88	-233	2.56E-02
		4	0.83	-206	2.79E-02
**Lysimeter**	**Linear**	1	0.64	−352	0.17
		2	0.44	−772	0.11
		3	0.56	−231	0.19
		4	0.85	−360	0.16
	**Segmented**	1	0.49	−671	0.13
		2	0.70	−1527	0.05
		3	0.42	−766	0.12
		4	0.57	−630	0.12

The results shown in Fig. 3 and Table 3, observed for one genotype with its four replicates, were representative of both panels, which exhibited substantial variability between trials. Overall, genetic variations in the transpiration response were best represented by slope differences of linear regressions. Consequently, the slope of the linear model was retained as a quantitative descriptor of the response.

### 3.4 No unique solution to measure transpiration response in canopy contexts

To determine the most suitable approach for capturing genotypic variation in transpiration responses to ET_ref_ in plants forming a canopy, whether under high-tech phenotyping or lysimeter conditions, contrasting results were observed across experiments.

The transpiration response slopes varied significantly among genotypes in the high-tech phenotyping trial (p = 0. 04772, Table 4), although the low heritability (H^2^_Cullis_ = 0.17; Table 4) indicates a limited genetic contribution to trait variation. In contrast, the lysimeter trial in sorghum showed no significant genotypic variation for this trait. This pattern did not hold for pearl millet in the lysimeter experiment, where transpiration response slopes differed significantly among genotypes and exhibited moderate heritability (p < 2.2 × 10^−16^; H^2^_Cullis_ = 0.61). However, this trial also displayed the highest coefficient of variation for the trait across experiments (CV = 0.45 for sorghum, 0.46 for pearl millet).

**Table 4:** Descriptive statistical results for measured traits. Mean, minimum (Min), maximum (Max), Coefficient of Variation (CV), p-values of ANOVA (G, taking into account repetitions, weeks, sowing batch and/or block as random effects if significant), Cullis estimation heritability (H^2^) on transpiration (transpiration rate, TR, or canopy transpiration, Tr) response slope measured on three platforms (low-tech, high-tech, and lysimeter), root area and root-shoot ratio (low-tech), and TE_plant_ (lysimeter).

	Trait	Mean	Min	Max	CV	G	H^2^
**Sorghum**							
**Low-tech**	**Root area**	2528	359	5937	0.46	2.2E-16 ∗∗∗	0.96
	**Root Shoot ratio**	0.50	0.04	1.14	0.47	2.2E-16 ∗∗∗	0.98
	**Slope TR-ET_ref_**	7.66E-05	1.78E-05	1.39E-04	0.27	2.2E-16 ∗∗∗	0.81
**High-tech**	**Slope Tr-ET_ref_**	0.65	0.30	0.95	0.18	0.04772 ∗	0.17
**Lysimeter**	**TE_plant_**	1.96	0.68	3.08	0.23	3.946E-03 ∗∗	0.25
	**Slope Tr- ET_ref_ x K_c_**	3.66E-02	5.29E-04	7.59E-02	0.45	0.4995	---
**Pearl millet**							
**Low-tech**	**Root area**	4.80E+03	3.49E+02	1.29E+04	0.60	0.03569 ∗	0.22
	**Root–Shoot ratio**	0.55	0.13	1.13	0.36	5.6E-04 ∗∗∗	0.32
	**Slope TR-ET_ref_**	7.97E-05	2.22E-05	1.43E-04	0.28	2.2E-16 ∗∗∗	0.73
**Lysimeter**	**TE_plant_**	1.94	0.17	3.61	0.27	2.2E-16 ∗∗∗	0.60
	**Slope Tr - ET_ref_ x K_c_**	0.13	4.10E-03	0.29	0.46	2.2E-16 ∗∗∗	0.61

Consequently, the lysimeter method appeared suitable for evaluating transpiration responses to evaporative demand in large panels of pearl millet genotypes forming a canopy. However, this conclusion does not extend to sorghum assessed in a different year. In the latter case, the high-tech phenotyping protocol was more appropriate for detecting genotypic differences in transpiration response slopes, although its effectiveness was limited by low heritability. Therefore, both methods appeared suitable for assessing transpiration responses to evaporative demand in canopy contexts.

### 3.5 Low-tech protocol produced the most accurate assessment of transpiration response to evaporative demand

Except the transpiration response slope in sorghum lysimeter cited above, other traits showed substantial variation in both sorghum and pearl millet, with the distribution of genotypic variation approximating a normal pattern (Fig. S4).

The transpiration response slope to evaporative demand in low-tech trials was similar between species (mean 7.66 × 10^−5^ vs 7.97 × 10^−5^, respectively in sorghum and pearl millet, range 1.78 × 10^−5^^-^- 1.98 × 10^−4^ vs 2.22 × 10^−5^ – 1.43 × 10^−4^, Table 4). This variation was associated with high heritability in both panels (H^2^_Cullis_ = 0.73 in sorghum vs 0.81 in pearl millet), higher than the heritability observed on other platforms. In the high-tech phenotyping trial for sorghum, the transpiration response to evaporative demand had a mean value of 0.65, ranging from 0.30 to 0.95, with a lower coefficient of variation than low-tech trials (CV = 0.18, Table 4). In lysimeter, the transpiration response slopes were lower in case of sorghum than pearl millet (mean = 3.66E-02 vs 0.13, min = 5.29E-04 vs 4.10E-03, and 7.59E-02 vs 0.29) with a higher CV than low-tech trials (CV = 0.45 and 0.46 in sorghum and pearl millet lysimeter vs 0.27 and 0.28 in sorghum and pearl millet low-tech trials).

To answer the question (v) comparing the three outdoor protocols studied here, the low-tech protocol was the most accurate assessment to study transpiration response to evaporative demand in large germplasm panels as it presented the strongest heritable results and genetic variation for both crops and a coefficient of variation that is intermediate between the other two platforms. Of course, the transpiration response to evaporative demand in individual plant in the high-tech platform would also need to be tested.

In addition to the robust results for transpiration response slopes, the low-tech platform allowed robust measurement of root traits. The root-to-shoot ratio was slightly higher in pearl millet (0.55, range: 0.13–1.13, CV = 0.36) compared with sorghum (0.50, range: 0.04–1.14, CV = 0.47). Root traits varied among genotypes in both species, with strong genetic control in sorghum (root area H^2^_Cullis_ = 0.96; root-to-shoot ratio H^2^_Cullis_ = 0.98) and low to moderate heritability in pearl millet (root area H^2^_Cullis_ = 0.22; root-to-shoot ratio H^2^_Cullis_ = 0.32). In the lysimeter trials, TE_plant_ was comparable between the two crops (1.94 g biomass.kg^−1^ water in pearl millet vs 1.96 g kg^−1^ in sorghum; CV = 0.27 vs 0.23) although the heritability was higher for pearl millet than sorghum H^2^_Cullis_ = 0.60 vs 0.25).

### 3.6 Trait correlations are largely platform-dependent

**Fig. 4:**
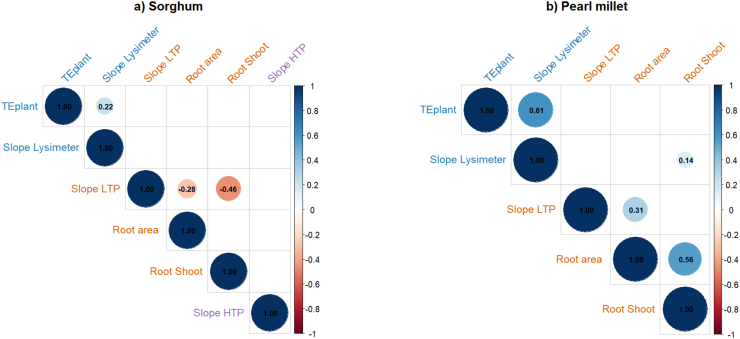
Correlation matrix of traits measured in the sorghum panel (left) and the pearl millet panel (right). Traits were measured in the lysimeter (blue), low-tech (orange), and high-tech (purple) platforms. Traits include: plant transpiration efficiency (TE_plant_) and “Slope Lysimeter”, the slope of the linear regression between transpiration (mm day^−1^) and reference evapotranspiration adjusted by the crop coefficient (ET_ref_ × K_c_, mm day^−1^) in the lysimeter; root-to-shoot ratio; root area; “Slope LTP” the slope of the linear regression between transpiration rate (g. h^−1^ cm^−2^) and ET_ref_ (mm h^−1^) in the low-tech platform; and “Slope HTP” the slope of the linear regression between transpiration (mm. h^−1^) and ET_ref_ in the high-tech platform.

In both crops, correlations were mostly observed among traits measured within the same experimental platform (Fig. 4). Overall, associations were stronger in pearl millet (Fig. 4, right) than in sorghum (Fig. 4, left). In sorghum, TE_plant_ measured in the lysimeters showed a weak positive though significant correlation with the slope of the transpiration response measured on the same platform (r = 0.22), whereas in pearl millet a similar and stronger relationship was found (r = 0.61). For low-tech measurements, the transpiration response slope was moderately negatively associated with root traits in sorghum, including root area (r = −0.28) and root-to-shoot ratio (r = −0.46), while in pearl millet, these associations were either positive (root area, r = 0.31) or weak (root-to-shoot ratio). Conversely, in pearl millet, root area was strongly and positively correlated with root-to-shoot ratio (r = 0.56), a relationship that was non-significant in sorghum.

Inter-experiment correlations were generally weak or absent. In particular, the transpiration response measured on the high-tech platform showed no significant association with traits from the lysimeter or the low-tech experiments in sorghum. The only minor inter-experiment correlation observed in pearl millet was between the lysimeter-measured transpiration response and the low-tech root-to-shoot ratio (r = 0.14).

## 4 Discussion

### 4.1 ET_ref_ -based variable are well-adapted to represent the evaporative demand

In our study, model performance improved (higher R^2^ and lower AIC and RMSE) when ET_ref_ -based variables (ET_ref_ or ET_ref_ × K_c_) were used instead of VPD, whereas most experimental studies of transpiration responses to the evaporative demand rely on VPD. In other words, in our outdoor phenotyping conditions, transpiration responses to the evaporative demand were better described when the descriptor incorporated additional atmospheric drivers such as solar radiation and wind speed.

Nevertheless, although this pattern was consistent across the three outdoor platforms analysed here, ET_ref_ cannot be regarded as universally more appropriate than VPD. The relative suitability of ET_ref_ versus VPD is likely context-dependent and influenced by geographic and climatic conditions. For instance, across the contiguous United States, the dominant drivers of extreme ET_ref_ exhibit strong regional variability, with air temperature prevailing in the northern regions, solar radiation in the south-eastern regions, wind speed in the southwestern regions, and combined temperature and humidity effects in the central regions US [34]. In environments where the evaporative demand is primarily controlled by temperature and/or humidity, or where radiation is high and above saturation for most of the day, VPD may therefore constitute a sufficiently robust and equally or more parsimonious descriptor than ET_ref_.

The choice of evaporative demand metric may also depend on experimental conditions and data availability. While VPD requires only air temperature and relative humidity, ET_ref_ was computed with the Penman–Monteith equation and required additional meteorological variables. In many regions, these variables are not routinely measured, not freely available, or are of limited quality due to insufficient quality control, resulting in reduced datasets that often lack solar radiation and wind speed [35]. Although approaches exist to estimate evapotranspiration from reduced datasets [36] or from alternative calculations methods such as the Hargreaves–Samani equation [37] or require modelling to work with air temperature alone [38], in such contexts, VPD remains a pragmatic way to represent the evaporative demand when more complete datasets needed for ET_ref_ are lacking.

### 4.2 Transpiration responses to evaporative demand outdoors were best fitted with linear regressions

Transpiration responses, represented by the relationship between transpiration and increasing ET_ref_, (or ET_ref_ x K_c_ in lysimeter trials) were best described by a linear model across the five experiments, and slope parameters provided a robust trait to compare genotypes. This result contrasted with the initial simulation study [2] and the subsequent experimental studies that reported nonlinear transpiration responses to VPD (e.g. Refs. [[4], [5], [6], [7], [8], [9],[11], [12], [13], [14], [15], [16], [17]]). However, our findings are consistent with those reported for maize by Alvarez-Prado et al. [18] and sorghum across contrasting canopy densities in lysimeter [10]. In that latter study, the authors attributed the absence of a breakpoint in transpiration response partly to the use of Penman–Monteith ET_ref_, which explicitly accounted for solar radiation as a major driver of transpiration. They further suggested that the linear response was related to the canopy-level nature of the measurements, as intra-canopy VPD increased less steeply than air VPD in lysimeter systems. Extending this interpretation, our results showed that even for individually spaced plants grown outdoors, as in our low-tech assessment, a linear model remained more appropriate than a segmented approach to describe transpiration responses to ET_ref_ under the same weather conditions and sandy soil than their sorghum lysimeter study. Taken together, these findings highlighted their first interpretation, with the dominant role of light in driving transpiration under outdoor conditions. The breakpoints found in growth chamber studies may be caused by an artefactual interaction between low light intensities and high VPD, i.e., the fact that transpiration stops increasing with VPD because of light limitation.

Although linear models performed best overall, the relative performance of linear versus segmented regressions varied among experimental platforms. Visual inspection of genotype-level responses indicated clearly that linear regression consistently provided the best fit in lysimeter experiments, whereas regression choice was less obvious in the other platforms, for instance in the high-tech platform where segmented regression also provided realistic breakpoints (contrary to segmented regression in lysimeter as comparison), and occasionally achieved slightly lower RMSE or AIC when looking at specific genotypes. More work would be needed on this topic because the two models (linear or linear segmented) represent different ways of stomata regulation, i.e. with a transient regulation in the case of a linear segmented model, in opposition to inherent stomatal conductance differences in the case of linear regressions.

### 4.3 Robust measurements of the transpiration response to ETref in the low-tech platform

A major outcome of this study is that phenotyping platforms strongly influence both trait expression and the detection of genetic variation. The low-tech platform consistently provided the highest signal-to-noise ratio, highest heritability, and most stable genotype ranking across traits in both species. These results demonstrated that a low-tech approach, relying on manual weighing, planimeter-based leaf area measurements, and substantial labour input over a short measurement window (a few days, compared with extended monitoring in lysimeter systems), could reliably quantify transpiration-related traits. The method captured strong genetic variation across large panels and generated heritable traits suitable for genetic analyses. This highlights the potential of low-cost phenotyping system as scalable tool that bridge the limitations of both high-tech and lysimetric platforms.

The high-tech platform showed the next best performance, benefiting from a large number of paired transpiration and evapotranspiration measurements with less constraining environmental conditions. This resulted in robust model fits and significant genotypic variation, although heritability estimate was comparatively low. This could be related to the difficult estimation of leaf area beyond 25 DAS in this platform, because of leaf overlap in plants forming a canopy. The high-tech platform was not tested to measure transpiration response of individual plant, in which we could expect similar heritability than in the low-tech platform, and much simpler operation.

In contrast, lysimeter experiments exhibited the highest level of noise in data fitting, with R^2^ values for transpiration response slopes generally lower than those obtained in the high-tech and low-tech platforms. The magnitude of this limitation differed between species: while pearl millet still exhibited significant genetic variation and moderate heritability, sorghum showed non-significant genetic effects. This poorer performance, particularly in sorghum, was likely driven by limited coverage of the ET_ref_ range. Linked with these ET_ref_ values ranges, the additional factor contributing to the differences between lysimeter and other experimental platforms was the frequency of measurements. In the high-tech and low-tech experiments, transpiration and ET_ref_ were estimated over four intervals within a day, allowing a broad characterization of evaporative demand. In contrast, lysimeter measurements were typically conducted every 3–4 days or more, resulting in temporal averaging of daily ET_ref_ variability. This averaging reduced the effective range of evaporative demand captured and likely contributed to noisier estimates of transpiration responses relative to the high-tech and low-tech platforms.

Together, these results demonstrate that phenotyping systems are not neutral measurement tools but actively shape the detectability of genetic variation in water-use traits.

### 4.4 TE_plant_ and transpiration response slopes are positively correlated in both crops

Despite the comparatively poorer model fits obtained in the lysimeter experiments, lysimeter-derived traits showed meaningful correlations in both crops. In particular, TE_plant_ was positively correlated with the transpiration response slope measured in the lysimeter, with a stronger association observed in pearl millet than in sorghum. The difference in the strength of this correlation between species may be related to environmental conditions, as ET_ref_ values were lower during the 2025 pearl millet experiment than during the 2024 sorghum experiment, potentially creating more favourable conditions for expressing this relationship. A species-specific effect cannot be excluded, however, and repetition of the pearl millet experiment would be required to disentangle environmental from genetic effects.

These findings are fully consistent with recent results reported by Pilloni et al. [10], who showed that TE_plant_ was positively correlated to the slope of the evapotranspiration response to ET_ref_ in seasons of high evaporative demand, which was also our case. This relationship for both crops in our current work could be interpreted as follows: under well-watered conditions, higher whole-plant transpiration reflects greater stomatal opening at the leaf scale, which enhances photosynthetic carbon assimilation. Higher TE_plant_ values could reflect a proportionally higher carbon assimilation than water loss. We do not have data to demonstrate this and can only interpret from the recent study from Pilloni et al. [10] and Vadez and Pilloni [39]. In the former study, genotypes combining high TE_plant_ and strong transpiration responses exhibited canopy architectures that allowed greater light penetration, notably through more erect leaf orientation, and lower VPD inside the canopy. Light penetration inside the canopy enhanced photosynthesis in lower canopy leaves, which experienced lower VPD than leaves directly exposed to the atmosphere, thereby contributing to higher TE_plant_. We speculate that the same phenomenon may have happened in our case.

### 4.5 Species-specific differences between transpiration response slopes to ET_ref_ and water uptake

Another notable intra-experiment relationship highlighted clear species-specific differences between root traits and transpiration dynamics in the low-tech trials. In pearl millet, larger root area was consistently associated with higher root-to-shoot ratios, indicating greater allocation of biomass to belowground organs. Larger root area was then associated with a higher transpiration response to ET_ref_. This correlation between transpiration and root area observed in well-watered conditions may reflect the development of an extensive root system to enhance soil water acquisition. Such coordination between root investment and transpiration response in pearl millet is consistent with a more opportunistic water-use strategy, whereby increased water uptake capacity supports higher transpiration per unit of leaf area as evaporative demand rises.

In contrast, sorghum exhibited a markedly different pattern. Root area was not related to higher biomass allocation belowground, and neither to higher root-to-shoot ratio. Moreover, greater root area and higher root-to-shoot ratios were associated with lower transpiration response to ET_ref_. This pattern may indicate that, a higher belowground investment in certain genotypes did not correlate with a higher response of the transpiration per unit leaf area to the evaporative demand, potentially reflecting tighter stomatal regulation. It could suggest possible species-specific differences in water-use strategies, with different coordination between root architecture and stomatal regulation in the two species.

The only weak inter-experiment correlation was observed between transpiration response slopes derived from the lysimeter and root traits in the low-tech pearl millet trial, both conducted in sandy soil. This finding aligns with preliminary work on a sorghum panel in sandy soil pots, which reported a slight positive correlation (r = 0.12) between root-to-shoot ratio and the slope of the transpiration response to ET_ref_, suggesting that higher root-to-shoot ratios were associated with steeper transpiration responses [40]. However, this relationship was not observed for sorghum in the present study.

### 4.6 Impact of the soil texture on the transpiration response slope to ET_ref_

Traits measured in the high-tech experiment conducted on clay soil and a high evaporative demand season showed no correlation with traits measured in sandy soils, i.e. in the lysimeter and low-tech sorghum experiments, also performed during a similar evaporative demand. We speculate that this could be caused by differences in soil texture, which is known to strongly influence plant water availability and uptake dynamics, and could modulate transpiration response slope to evaporative demand. These speculations are consistent with previous findings showing that soil properties significantly affect transpiration responses to increasing VPD [41]. Clay soils, characterized by high water-holding capacity would retain water more and reduce the rate at which plants can use up water, whereas sandy soils would tend to allow a faster water depletion. In line with this, soil texture has also been identified as a key driver of variation in plant transpiration efficiency. For example, sorghum and maize genotypes have been reported to exhibit lower TE_plant_ in sandy soils compared with clay-rich vertisols [42], a pattern hypothesized to arise from differences in transpiration regulation across soil types.

Taken together, these results suggest that soil texture likely contributed to the observed variation in transpiration response slopes across trials. Future experiments would therefore benefit from the use of consistent soil textures across platforms, both to enable more robust comparisons of transpiration-related traits and to assess potential links between responses measured in high-tech and low-tech platforms.

## 5 Conclusions and perspectives

By integrating multiple outdoor phenotyping platforms, this study demonstrates that, using short time frames to measure transpiration, reference evapotranspiration derived from the Penman–Monteith equation, combined with a linear modelling approach offers a robust protocol to quantify and pinpoint genetic variation in the transpiration response to evaporative demand for both individual plants and plants forming a canopy. Although we were not able to conclude on a unique solution to measure canopy transpiration response to evaporative demand, the low-tech outdoor phenotyping with individual plants coupled with short timeframes provided the most reliable and heritable trait estimates, underscoring its potential as a cost-effective solution for large-scale phenotyping and for supporting breeding programs targeting improved water-use strategies in resource-limited environments. Species-specific differences further suggest contrasting water-use strategies between sorghum and pearl millet, shaped by distinct physiological coordination between transpiration, water-use efficiency, and root architecture. These findings highlight the need for further work to disentangle the coordination between below- and above-ground processes underlying adaptation to atmospheric drought.

## Ethics approval and consent to participate

Not applicable.

## Consent for publication

Not applicable.

## Availability of data and materials

The data that support the findings of this study are available from the first author upon reasonable request. All scripts used for analyses, as well as for reproducing the results and figures presented in this study, are publicly available on GitHub:

https://github.com/lcmgregoire/ANALYSIS_PHENOTYPING_TR_ETREF.

Modifications, including corrections and improvements implemented in the modified Kar et al. pipeline, are also publicly available on GitHub:

https://github.com/lcmgregoire/UPDATE_KAR_PIPELINE_LEASYSCAN_EZTr.

## Authors' contributions

LG contributed to Conceptualization, Methodology, Formal analysis, Data curation, and Writing (original draft). JK, SC, CD, and VV contributed to Conceptualization and Methodology. SC and RB contributed to Investigation on the high-technology Indian platform. LG, JN, CD, OS, and CS contributed to Investigation on the low-technology platform, with OS and CS contributing to Investigation and Data curation through root trait measurements. RB contributed to Investigation on the lysimeter platform. VV and YV contributed to Supervision, with oversight of Data analysis and Writing (review & editing).

## Funding

The senior and first corresponding author are supported by a grant the Make Our Planet Great Again (MOPGA) ICARUS project (Improve Crops in Arid Regions and Future Climates) funded by the French Agence Nationale de la Recherche and by the Region Occitanie (ANR, grant ANR-17-MPGA-0011).

## Declaration of competing interest

The authors declare that they have no known competing financial interests or personal relationships that could have appeared to influence the work reported in this paper.

## List of abbreviations

ET_ref_: reference evapotranspiration
VPD: Vapor Pressure Deficit
TE_plant_: Transpiration efficiency at plant level
DAS: days after sowing
AIC: Akaike Information Criterion
RMSE: Root Mean Square Error
BLUP: Best linear Unbiased Prediction
