# Supplementary material for: Transpiration responds linearly to Penman-Monteith reference evapotranspiration and varies genetically, both in individual plants and canopies, in large sorghum and pearl millet panels
Source: Plant Phenomics. 2026 Jun 3;8(2):100231. doi: 10.1016/j.plaphe.2026.100231 (PMC13293747; doi:10.1016/j.plaphe.2026.100231)
Supplement: Multimedia component 1 [file mmc1.docx]

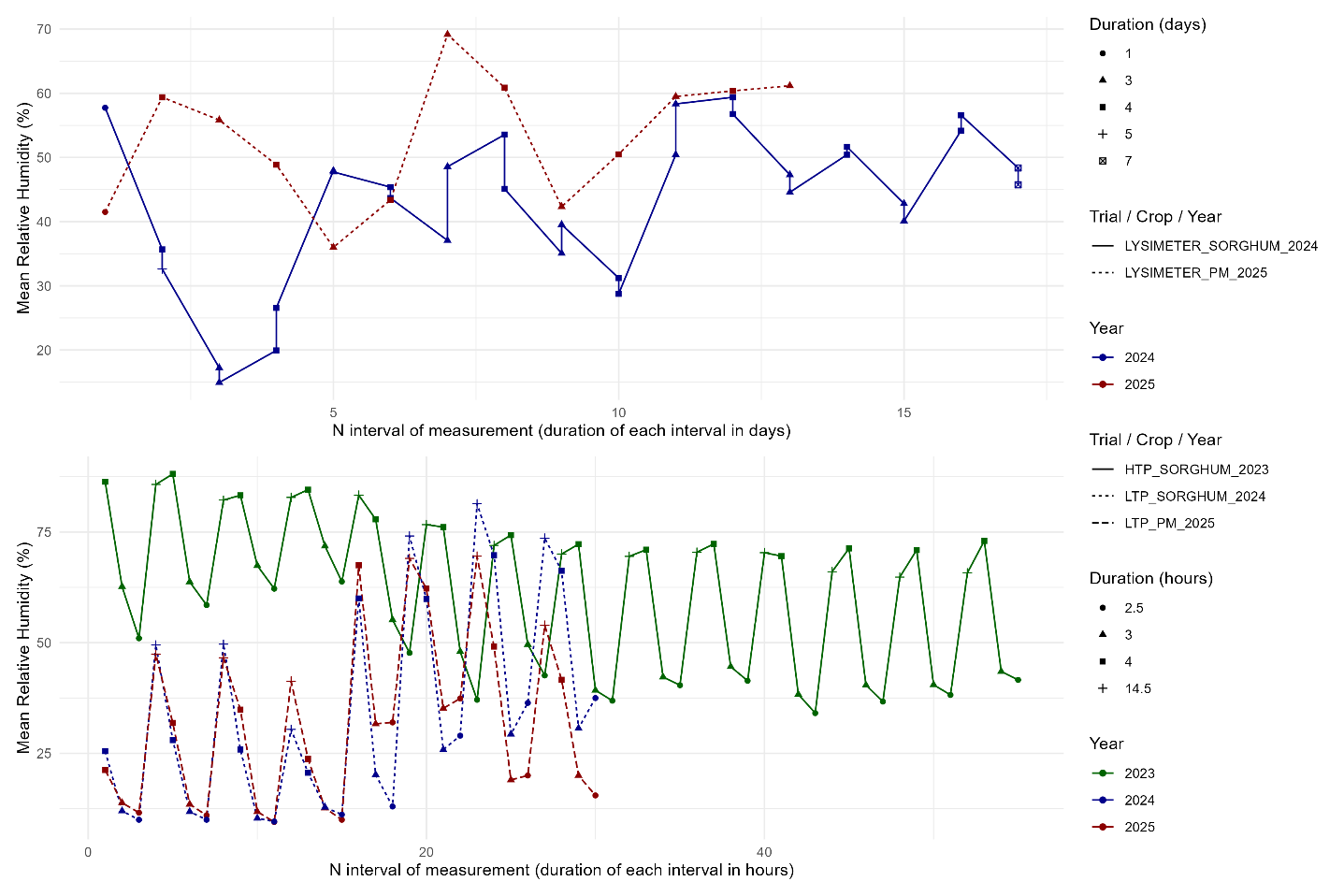


Figure S1 Mean relative humidity (%) across the studied time intervals for the lysimeter experiments (top panel) and the high- and low-tech experiments (bottom panel). Experiments conducted in 2023 (high-tech) are shown in green, those conducted in 2024 (lysimeter and low-tech sorghum experiments) in red, and those conducted in 2025 (lysimeter and low-tech pearl millet experiments) in blue. The duration of each interval is indicated by different symbols (days for the lysimeter experiments and hours for the HTP and low-tech experiments).


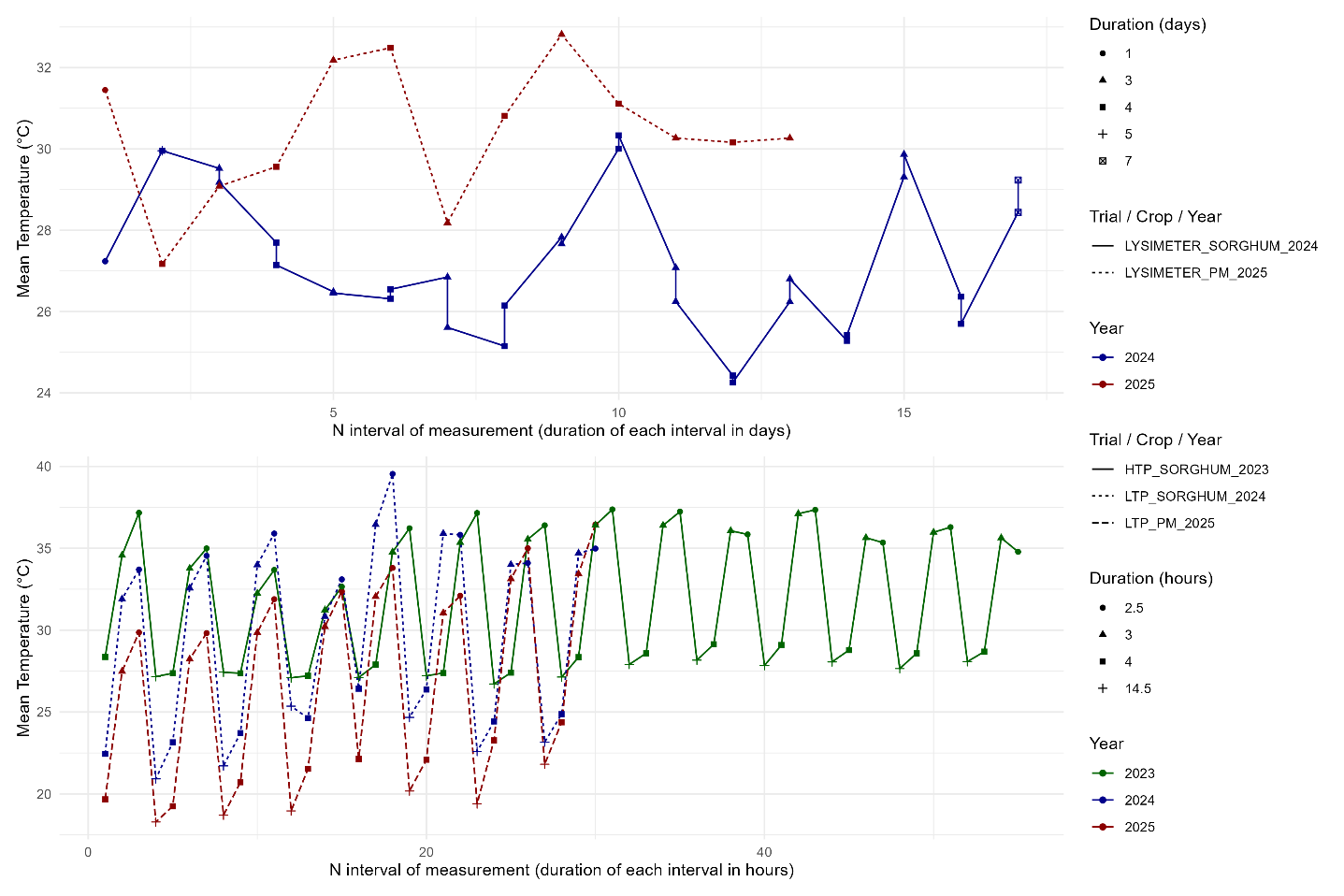


Figure S2 Mean temperature (°C) across the studied time intervals for the lysimeter experiments (top panel) and the high- and low-tech experiments (bottom panel) Experiments conducted in 2023 (high-tech) are shown in green, those conducted in 2024 (lysimeter and low-tech sorghum experiments) in red, and those conducted in 2025 (lysimeter and low-tech pearl millet experiments) in blue. The duration of each interval is indicated by different symbols (days for the lysimeter experiments and hours for the HTP and low-tech experiments).


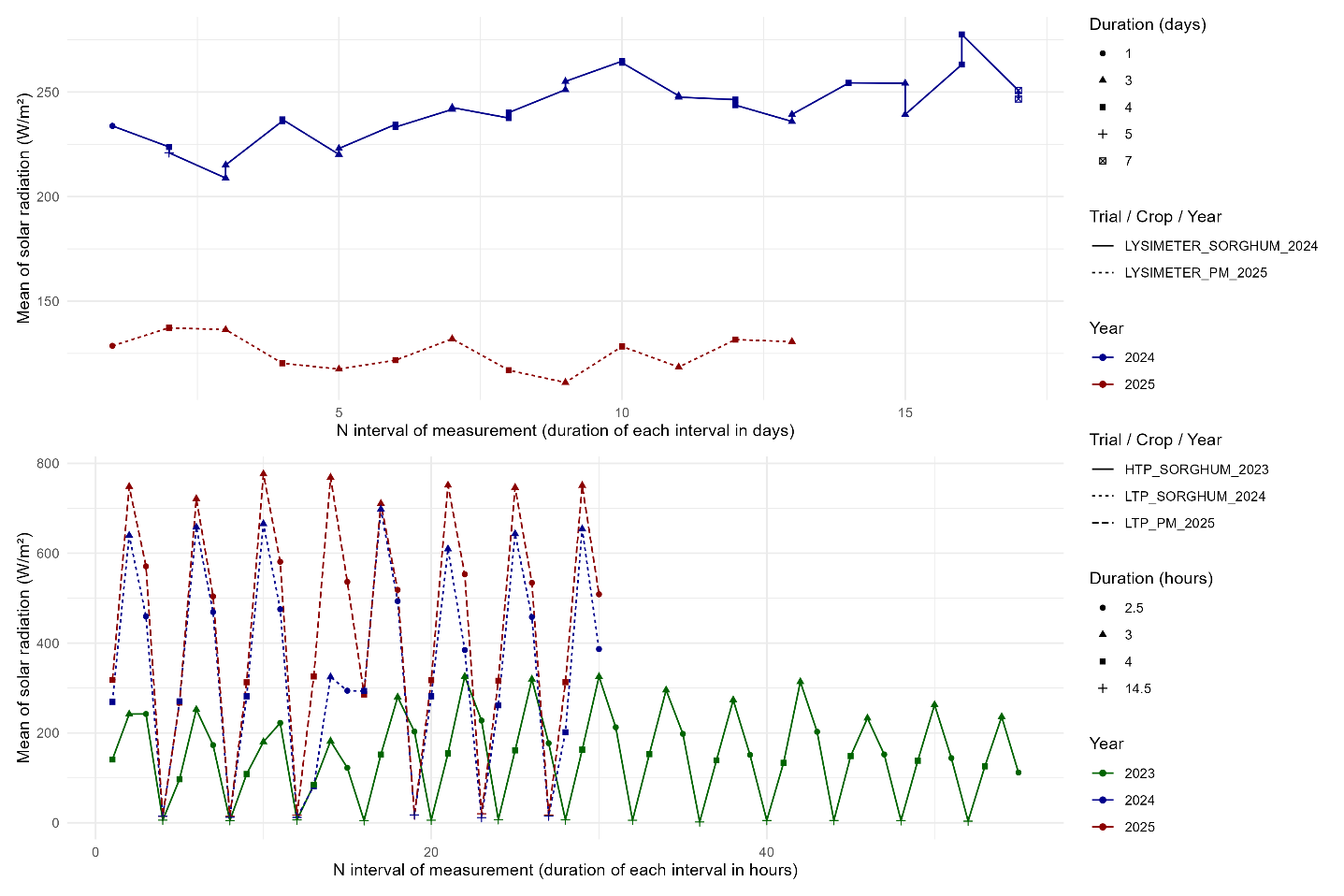


Figure S3 Mean solar radiation (W/m²) across the studied time intervals for the lysimeter experiments (top panel) and the high- and low-tech experiments (bottom panel). Experiments conducted in 2023 (high-tech) are shown in green, those conducted in 2024 (lysimeter and low-tech sorghum experiments) in red, and those conducted in 2025 (lysimeter and low-tech pearl millet experiments) in blue. The duration of each interval is indicated by different symbols (days for the lysimeter experiments and hours for the HTP and low-tech experiments).


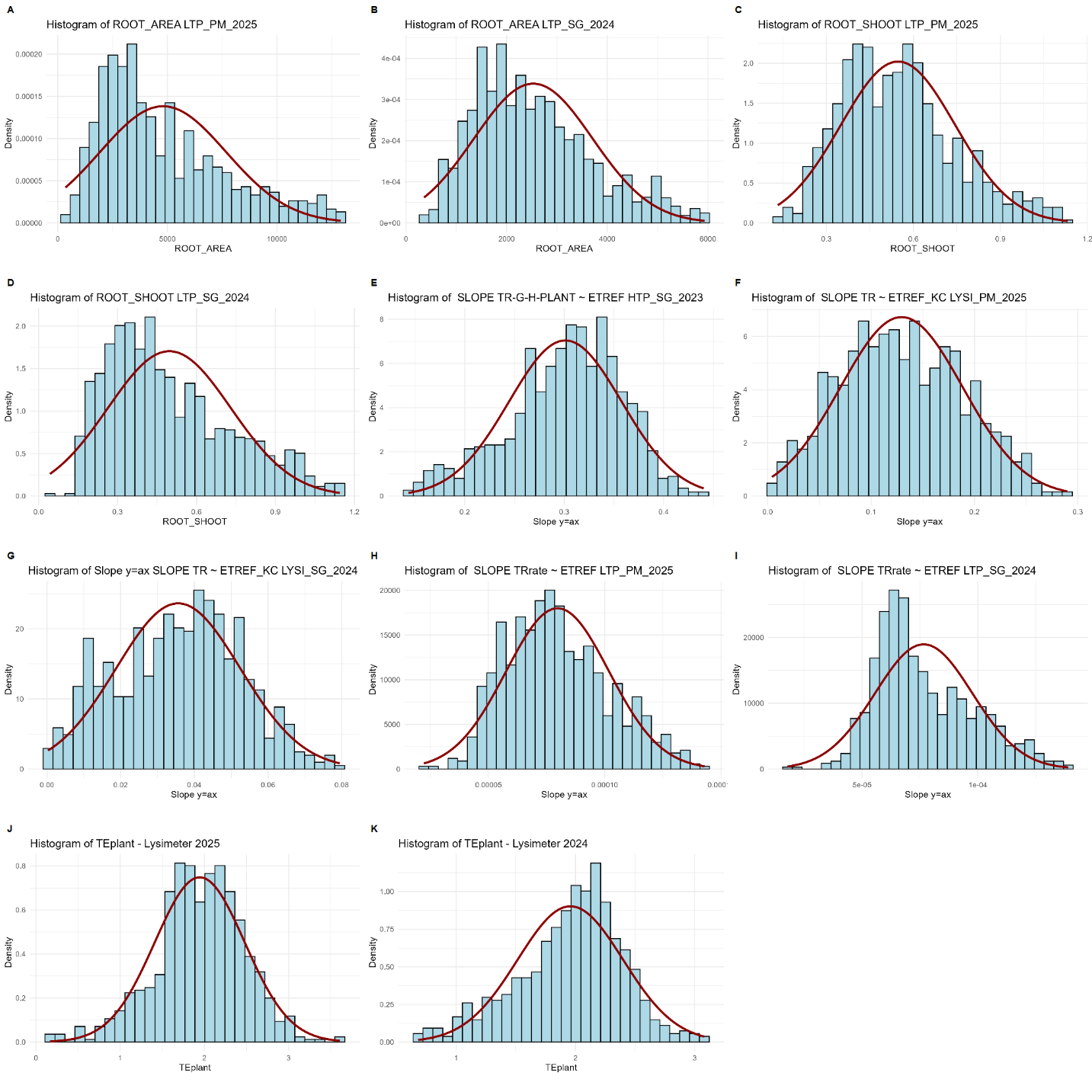


Figure S4 Frequency distributions of the 11 variables studied across five trials involving two crop species. The variables include: slope measured using High-tech Phenotyping Platform in sorghum; slope, root-to-shoot ratio, and root area measured using Low-tech Phenotyping Platform in both crops; and TE_plant_ and slope measured using lysimeter systems in both crops. All traits presented normal distribution.
